# ‘I don’t consider cancer when I’m grabbing the beer’: Discursive strategies used by midlife New Zealanders to undermine alcohol-cancer risks

**DOI:** 10.1093/heapro/daaf184

**Published:** 2025-11-06

**Authors:** Antonia C Lyons, Kate Kersey, Jessica Young, Christine Stephens, Denise Blake, Ragnar Anderson

**Affiliations:** Centre for Addiction Research, Department of Social and Community Health, University of Auckland, Private Bag 92019, Auckland 1142, Aotearoa New Zealand; Centre for Addiction Research, Department of Social and Community Health, University of Auckland, Private Bag 92019, Auckland 1142, Aotearoa New Zealand; Centre for Addiction Research, Department of Social and Community Health, University of Auckland, Private Bag 92019, Auckland 1142, Aotearoa New Zealand; School of Health—Te Kura Tātai Hauora, Victoria University of Wellington, PO Box 600, Wellington 6140, Aotearoa New Zealand; School of Psychology, Massey University, Private Bag 11-222, Palmerston North 4442, Aotearoa New Zealand; Independent Researcher, Wellington, Aotearoa New Zealand; School of Health—Te Kura Tātai Hauora, Victoria University of Wellington, PO Box 600, Wellington 6140, Aotearoa New Zealand

**Keywords:** alcohol, health risk, cancer, discourse, qualitative methods

## Abstract

Compared with other age groups, adults at midlife consume alcohol at relatively high levels. Alcohol has been linked to a number of long-term health risks, including cancer, although awareness of cancer risk is low. The current study aimed to examine how adults at midlife talk about, understand and consider alcohol-related cancer risks within their life contexts. Individual interviews were undertaken with 37 adults (41–64 years; 28 female, 9 male) about their alcohol consumption, views on the health risks of drinking, and understandings of the alcohol-cancer association. Interviews were transcribed verbatim, coded and subjected to a discursive analysis. Participants constructed their drinking as low-risk because it was controlled, responsible, and moderate. They used discursive strategies to undermine the evidence on the cancer risks of alcohol by contrasting it with (stronger) evidence for tobacco risk, drawing on personal accounts of exceptional cases, and displaying ‘risk fatigue’ because alcohol was just one of many carcinogens they navigate in daily life. The pleasure they derived from alcohol outweighed cancer risks. Cancer risk evidence was itself constructed as risky because people with cancer could be blamed for their disease. These findings show that public health messages about alcohol and cancer risk need to incorporate people’s own sense-making about alcohol and risk within their lives, including notions of pleasure. Unintended consequences of current messaging include short-term risks (to health and wellbeing) and moral risks (potential for people to be blamed for cancer) and therefore may be ignored or resisted by target populations.

Contribution to Health Promotion

- Alcohol consumption has been linked to a number of long-term health risks, including cancer
- Midlife adults have relatively high levels of alcohol consumption, but low levels of awareness of cancer risks. How adults at midlife make sense of alcohol-related cancer risks is important.
- Midlife adults undermined evidence on cancer risks of alcohol using a range of discursive strategies and identified factors that outweighed the cancer risks of drinking. They also viewed accepting the evidence on alcohol and cancer risk as itself risky.
- Health promotion messages need to incorporate people’s own sense-making about alcohol and risk within their lives.

## INTRODUCTION

Rates of alcohol consumption in midlife and older adults continue to be relatively high, despite declining rates among teenagers in countries such as Aotearoa New Zealand (NZ), Australia, and the United Kingdom (UK). In NZ, ∼18% of midlife adults (35–44 years) and 22% of older adults (45–54 years) reported drinking at hazardous levels in 2023 (Ministry of Health 2024). Similarly, approximately a third of Australian adults aged 30–59 years reported drinking more than the government’s low-risk drinking guidelines (Australian Institute of Health and Welfare 2024) and 12%–14% of adults in the UK aged 35–54 years reported drinking hazardously (NHS England 2024). These levels of drinking are concerning given the evidence demonstrating links between alcohol consumption and increased risk for many cancers, including mouth, throat, oesophagus, liver, colon, rectum, and breast cancers (Connor 2017, Connor *et al*. 2017, Rumgay *et al*. 2021). Evidence suggests that any alcohol consumption increases cancer risk, with risk rising as consumption amounts increase (Paradis *et al*. 2023). Yet public awareness of this risk is relatively low. In NZ, only 18% of adult survey respondents spontaneously stated that alcohol is related to cancer risk (Peniamina *et al*. 2023), while in Australia only 17% of adults surveyed mentioned cancer when asked about the health risks of alcohol (Tabbakh *et al*. 2021). In the UK, 45% of adult survey respondents listed alcohol use when asked what things might increase cancer risks (Whitelock 2023).

Health promotion initiatives use education campaigns and government low-risk drinking guidelines to inform the public about the long-term risks of alcohol. Guidelines are often expressed as reducing short- and long-term health risks by not exceeding a maximum number of drinks per week. However, education campaigns aimed at increasing awareness of the cancer risks associated with alcohol have been found to increase people’s knowledge, attitudes and beliefs about alcohol but not reduce consumption levels (Young *et al* 2018, Meyer *et al*. 2019, Booth *et al*. 2023). In Australia, only 57% of adult survey respondents who were aware of the alcohol-cancer link said this motivated them to drink less (FARE 2020). In NZ, only 28% of survey respondents stated that knowledge about the alcohol-cancer link motivated them to drink less (Lyons *et al*. 2025).

Disseminating expert knowledge to people about behaviours that increase their cancer risk does not necessarily, or automatically, lead people to change their behaviour to reduce their risk (Kelly and Barker 2016). In contemporary Western societies, the public is informed of a multitude of open-ended risks for poor health outcomes based on expert, scientific and technical knowledge, knowledge which shifts and changes as it is subject to contestation and revision by experts. This has led to scepticism of risk science generally (Beck 1992, Lupton 2023) that intensified throughout the COVID-19 pandemic (Alaszewski 2003, Caplan 2023). Importantly, people engage with, reflect on, and make sense of risks within their own belief systems and moral positions which shapes their actions (Lupton 2023). Understandings and responses to risk information are embedded within people’s social contexts, with economic, social, cultural, and emotional factors contributing to a personal sense of risk and the desire and ability to act (Meyer *et al*. 2019, Lunnay *et al*. 2021a). These ‘lay epidemiology’ factors shape a personal sense of ‘candidacy’, the extent to which someone sees themselves as likely to get cancer and thus willing to respond to risk information (Ward *et al*. 2022, Foley *et al*. 2024b). Adults in the UK have been found to use lay epidemiology to explain the impact of their drinking and its health risks (Lovatt *et al*. 2015, Larsen *et al*. 2023). How people experience and understand alcohol consumption (and its risks) is social, cultural, and gendered (Lyons *et al*. 2014).

Qualitative research has explored the ways in which people understand and make sense of the cancer risks of drinking alcohol. Researchers found that midlife women in Australia are distrustful and uncertain about the scientific information on the breast cancer-alcohol link, have doubts about their candidacy for breast cancer, and emphasize the roles and functions of drinking alcohol (Foley *et al.* 2024b, 2024c). Younger women in Australia did not view the risk of breast cancer from drinking alcohol as something that was temporally relevant to them, rather something to worry about later in life (Foley *et al.* 2024b, 2024c). These authors discuss ‘immediate risk horizons’ as a crucial aspect of how people make sense of and consider minimizing risk. Other research shows how Australian adults question the legitimacy of having cancer warning labels on alcohol products by using discursive strategies to undermine the scientific credibility of the evidence, counter public health messages, and be sceptical about risk information (May *et al*. 2022).

Public health risk messaging sits alongside research showing that many midlife adults position drinking alcohol as a pleasurable activity that involves altered embodied states (Lunnay *et al*. 2022, Lyons *et al*. 2014, 2023, Keane 2023). Indeed, women at midlife construct drinking alcohol for a range of reasons, including pleasure, friendship, sociability, relaxation and coping (Lunnay *et al.* 2021a, 2023, Kersey *et al.* 2022, Keane 2023). Further, those with fewer resources, and those who experience midlife as a time of significant pressures and life transitions, may orient more strongly towards alcohol consumption (Lunnay *et al*. 2021a, Lunnay *et al*. 2023, Davies *et al*. 2024, Patsouras *et al*. 2025). The meanings and values that midlife adults ascribe to their drinking, and their embodied experiences of alcohol consumption, are all key factors that determine when and how they drink, rather than considerations of health risks (Lyons *et al*. 2014, Kersey *et al*. 2022, Larsen *et al*. 2023).

Public health experts argue that we need to increase people’s awareness of the cancer risks of alcohol consumption so that they can make informed decisions about their drinking. However, as discussed, research shows that even when people are aware of the evidence they do not change their drinking behaviour. We currently do not know why awareness of alcohol as a carcinogen often does not lead to drinking less. This study aimed to examine how adults at midlife in NZ talk about, understand and consider the cancer risks associated with alcohol consumption in their life contexts. It drew on notions of risks as embedded within everyday lives, as socially and culturally mediated, and as situated within broad ideas circulating in society (Lupton 2023) to explore the discourses (shared sets of meaning) and discursive strategies that adults at midlife used to construct drinking and notions of cancer risks in their talk.

## METHODOLOGY

The study was situated within a social constructionist paradigm and employed in-depth individual interviews with adults at midlife. Ethics approval was granted by Victoria University of Wellington Human Ethics Committee.

### Recruitment

Participants had previously taken part in an online survey (6–10 months earlier) about alcohol, drinking practices and awareness of health risks and had indicated they were interested in being interviewed. The survey used a convenience sample of midlife adults (aged 40–65 years) recruited through social media, personal and professional networks. They had to be living in NZ and drink alcohol at least once a week. Respondents came from towns and cities across NZ; there were more females (70%) than males (28%) or other genders (1%) (see Lyons *et al*. 2025). Those who volunteered to be interviewed were contacted by email.

### Participants

Thirty-seven participants took part in individual interviews. As shown in Table 1, they were aged 41–64 years (M = 45.2) and most were female (reflecting the survey sample), heterosexual, married, parents and Pākehā/European; two identified as Māori and one as Pacifica. Two-thirds were regular drinkers, consuming alcohol a few times each week. Over 60% said they had only one or two drinks when they drank alcohol, with 25% saying they had 3–4 drinks.

**Table 1.** Participants’ demographic and alcohol consumption information (*N* = 37).

Participant information	Categories	N	%
**Gender**	Female	28	77.8
	Male	9	25.0
**Age**	41–45	11	30.6
	46–50	4	11.1
	51–55	11	30.6
	56–60	6	16.7
	61–64	4	11.1
**Primary ethnicity**	Pākehā or European	33	91.7
	Māori	2	5.6
	Pacifica	1	2.8
	Did not say	1	2.8
**Socioeconomic status (based on household income^a^)**	Low	7	18.9
	Medium	14	37.8
	High	13	35.1
	Did not say	3	8.1
**Sexuality**	Heterosexual	35	97.2
	Bisexual	1	2.8
	Lesbian	1	2.8
**Location**	Large city	29	81.6
	Small-medium towns	8	22.2
**Current relationship status**	Married/de facto	28	77.8
	In a relationship	2	5.6
	Single	2	5.6
	Did not say	5	13.9
**Current parent status**	Children living at home**^b^**	21	58.3
	Children not living at home	9	25.0
	Not a parent	6	16.7
	Didn’t say	1	2.8
**Usual frequency of drinking alcohol**	Less than once per month	3	8.3
	A few times a month	4	11.1
	A few times (2–3) a week	24	66.7
	More than 3 times a week	5	13.9
**Usual amount consumed on one occasion**	1–2 drinks	22	61.1
	3–4 drinks	9	25.0
	5–6 drinks	2	5.6
	7–9 drinks	2	5.6
	10+ drinks	1	2.8

^a^Using household income bands from Statistics NZ, 2022. ^b^Includes step-children and one grand-child.

### Procedure

Interviews were conducted online (*n* = 34; 92%) or in person (*n* = 3; 8%) throughout 2022. A semi-structured interview guide asked participants about the roles and meanings of alcohol in their lives, drinking practices, views on the longer-term health effects of drinking, and understandings of the links between drinking and cancer risks. Interviews lasted between 30–60 minutes and were audio-recorded. Participants received a NZ$20 supermarket voucher. Interviews were transcribed verbatim and de-identified. All participants’ names are pseudonyms.

### Analytic approach

We undertook a Foucauldian-informed discourse analysis (Burr and Dick 2017, Phillips 2023), an approach that investigates how knowledge and meaning is constructed and maintained, and what this can enable and constrain (Riley *et al*. 2021, Phillips 2023). It seeks to identify discourses and discursive formations, the subject positions enabled by particular constructions, and consider their implications. Discourse refers to a historically located system that produces knowledge and shared meanings; discourse shapes how topics are meaningfully spoken about and how people understand an issue (Riley *et al*. 2021). Some discourses become so familiar and entrenched that they take on the status of truth, naturalizing specific understandings as ‘how things are’.

We first coded transcripts using NVivo, focusing on drinking practices, roles, and functions of alcohol, views on short- and longer-term health risks, and the association between alcohol and cancer. Codes were then combined into broader groupings, which we examined for contradictions and tensions, as these reveal dominant discourses used by participants to smooth over inconsistencies in their talk. Throughout analytic discussions we examined how participants constructed particular objects (alcohol, drinking), the subject positions that arose from constructions, and their potential implications.

### Researcher positionality

The research team included two early-career, one mid-career, and two senior researchers, as well as two research assistants (postgraduate students who undertook most of the interviews). We are all women aged across early, middle, and late midlife. Six of us identify as Pākehā (NZ European) and one as Māori; six grew up in NZ and one had moved to NZ from the USA. Five of us drink alcohol, while two no longer drink. We are all highly educated, middle-class parents (with young and adult children) and familiar with living in NZ society where alcohol and its consumption are normalized and expected. Our positionings were valuable for identifying the specific discourses and constructions participants drew on. We discussed the data from our different cultural and kinship backgrounds, life and family stages. Our focus was on broader societal discourses that both males and females used when talking about alcohol and drinking. Analysis was led by a senior and an early career researcher.

## FINDINGS

Throughout their talk, participants drew on three dominant discourses. They used a ‘healthism*’* discourse in which maintaining good health is positioned as the individual’s responsibility, and citizens have a moral imperative to behave in ways that keep them healthy (Crawford 2006). They were keen, perhaps understandably given the research context, to position themselves as responsible citizens making sensible choices about alcohol, and used a ‘risk’ discourse to achieve this. They also drew on a ‘moderate, responsible drinking’ discourse in which they constructed themselves as moderate drinkers who enjoy alcohol responsibly. Together these three discourses enabled participants to position their alcohol use as normative, mundane, responsible, and considered, similar to previous findings (Gough *et al*. 2020). Below we first briefly describe how participants used these discourses to position their drinking and cancer risk, and then focus on three discursive strategies they used to question (and resist) the alcohol-cancer risk.

### Healthism, risk and moderation discourses: constructing responsible and health-aware drinkers

Participants were thoughtful about their alcohol use within the context of a risk society (Beck 1992) in which we expect citizens to comply with behavioural advice—based on scientific and medical evidence—to reduce their risks of long-term diseases through modifying diet, exercise, smoking, vaping, amount of sleep, and so on. All participants constructed their drinking as restrained, controlled and not enough to be a risk for cancer:

I don’t think about that, because I don’t think I drink enough for it to be of concern. Nadia, 53 years; consumes 1–2 drinks, 1–2 times/week

No, it probably doesn’t worry me, and I probably don’t think about it, because in my mind, rightly or wrongly, I see my alcohol consumption as light to moderate. Simon, 47 years; consumes 4 drinks, 1–2 times/week

Nadia and Simon both described their alcohol use as relatively light and also claim that these (low) levels would not increase their risk of cancer, although both also hedged these claims using tentative words, such as ‘I don’t think’, ‘probably’ and ‘rightly or wrongly’. This hedging suggests they could be presented with evidence to the contrary, implying (their) current knowledge may not be up-to-date and that knowledge is changing.

In the following quote, Edward also attempts to claim that his level of drinking would not affect his cancer risk, although he is not sure:

But for me in my current stage of life I know drinking is bad, and drinking regularly is bad, but I make the naïve assumption that my level of drinking won’t cause too much for a possibility of getting cancer because of it. Um, I don’t know if I’m just being self-delusional, or if I’m being pragmatic, I don’t know. But [in] my day to day I don’t consider cancer when I’m grabbing the beer or the baileys on ice or anything like that. Edward, 45 years; consumes 2 drinks, twice a week

Here, Edward draws on a ‘healthism’ discourse to demonstrate his knowledge that drinking—and drinking regularly—are ‘bad’ and positions himself as responsible by drinking at low enough levels to not risk getting cancer. Yet, he is not sure about where his level of drinking actually sits within the ‘risk’ continuum, and positions himself as potentially blind to the risks (‘a naive assumption’, ‘self-delusional’). Simultaneously, he positions his drinking as mundane, part of his ‘day-to-day’, implying it is restrained and ordinary and unlikely to be related to cancer risk.

As Edward’s quote demonstrates, continuing to drink when knowing about the cancer risks required some discursive manoeuvring. This was also apparent in other participants’ talk. Below, we describe how participants unsettled the alcohol-cancer association through de-legitimizing the evidence, drawing on exceptional cases, and emphasizing how everything causes cancer in modern life. These strategies enabled participants to position their doubting of the cancer risks of alcohol use as logical and commonsense, thereby justifying their continued responsible drinking. Participants also constructed additional risks that arose should they accept alcohol-related cancer risks, including risks to their own wellbeing and moral risks for themselves and people with cancer who might be judged and blamed for the disease.

### Unsettling the evidence: it is logical to doubt the claim that drinking alcohol increases cancer risk

Participants used a number of discursive strategies to question the legitimacy of the evidence showing alcohol consumption leads to increased risk of cancer. They contrasted the stronger evidence of a direct link between tobacco and cancer; they drew on exceptional cases to question the link; and they employed dominant understandings of risk in a world where ‘everything causes cancer’. These are outlined below.

#### De-legitimizing the evidence by contrasting with tobacco evidence

Some participants contrasted the evidence linking tobacco to cancer with that linking alcohol to cancer in a way that questioned the certainty of the alcohol-cancer association and undermined the scientific evidence. For example, Daphne can ‘imagine’ the link between alcohol and cancer, although compared with cigarettes it is not strong:

I imagine there is a link…[but it’s] not like I would have with cigarettes. Daphne, 43 years; consumes 3–4 drinks, 2–3 times/week

In the quote below Jody draws on body parts and specific types of cancer to construct a direct link between smoking and cancer and an indirect link between drinking and cancer:

… because lung cancer is associated with smoking but what cancer is associated with drinking? It’s not, it's not a direct link. Jodie, 52 years; consumes 2–3 drinks, 4 + times/week

Jodie questions the legitimacy of the drinking-cancer link because no specific cancer is associated with drinking. Simon discussed how being an ex-smoker was a greater concern for him around cancer risk than drinking alcohol:

I probably worry more about the fact that I’m an ex-smoker than I ever would about um drinking in relation to cancer. …. I would think about that 50 times more than I would about the link between alcohol and cancer. Simon, 47 years; consumes 4 drinks, 1–2 times a week

By contrasting smoking and drinking, and using a number (50 times) to emphasise his point, Simon constructs the link between alcohol and cancer as less certain and therefore something he worries less about.

#### Using exceptional cases to show the imprecision of population risks

Some of the participants also undermined the link between alcohol consumption and cancer through drawing on exceptional cases, often personally known to them. These cases were used discursively to highlight uncertainty around population-level risks and the imprecision of the evidence that drinking alcohol increases cancer risk. For example, Amanda and Jodie talked about friends with cancer, both of whom did not drink:

But yeah, this one friend, and I find it quite shocking, because she’s already had bowel cancer right ….but she wasn’t a big drinker…[later] And a really good friend of mine, she’s super healthy, she runs and she wasn’t even a big drinker or anything, and she’s got breast cancer. Amanda, 53 years; consumes 2 glasses of wine, 2–3 times/week

actually my two girlfriends that have breast cancer and passed, neither of them drank at all. Jodie, 52 years; consumes 2–3 drinks, 4+ times/week

Andrea also talked about a friend who got cancer who did not drink:

Well, no, I mean, cancer such a, such a bad luck, like my friend right, some people live, amazingly healthy lives, don’t smoke, don’t drink, don’t do anything; bed eight hours sleep every night, and they are riddled with that. Yeah and then other people like, live dangerously and do not get it so yeah. Andrea, 44 years; consumes 2 drinks, 1–2 times/week

Here, Andrea constructs cancer as ‘bad luck’, random, and not related to behaviours such as drinking. She justifies this construction by providing a long list of healthy behaviours that her friend, and more generally ‘some people’, engage in, and yet who still get cancer. She strengthens this construction by describing the opposing spectrum of people, those who ‘live dangerously’—engage in unhealthy behaviours—but who do not get cancer. This functions to question the alcohol-cancer link.

Megan similarly described people she knew who had ‘abstemious’ lives, including those who ‘hardly drink’, but who have died from cancer:

As far as health and cancer goes and touch wood I’ve been fine, um, I think, having people my age die who led much more abstemious lives than myself certainly has not swayed me to drink less because I’m thinking [high voice] you hardly drink, you, do that and look at y- and you died so mmm. That is part of my headset of all this. That’s why I don’t worry. Megan, 62 years; consumes 5–6 glasses of wine once a weekend, less during the week

Megan uses personal experiences of people getting cancer who have lived healthy lives as a justification for not drinking less, and therefore also not worrying about her drinking. Outlining exceptional cases, people who get cancer even when they engage in healthy lifestyles, which include not drinking or drinking very little, functions to de-legitimise the alcohol-cancer link.

#### Emphasizing that ‘everything causes cancer’—risk fatigue

Some of the participants employed commonsense understandings that within our lives, societies and worlds, everything causes cancer. This explanation minimised the alcohol-cancer risk because it is just one of many risks that people are responsible for navigating in everyday life. For example, Jessica states:

It’s more the short-term that I’m worried about to be honest, because I feel like. Especially with cancer, I mean no one’s immune right, you can get cancer from anything if it’s not one thing it’ll be another, it seems, so I think it’s pretty hard to avoid. Jessica, 44 years; consumes 1–2 drinks once a month

Jessica draws on temporal notions of risk and constructs cancer as a long-term risk, something that might happen in the future, from any number of sources, in contrast to the short-term risks she is worried about now. Jessica’s talk functions to negate any suggestion that people should reduce their alcohol consumption to avoid cancer by creating a convincing three-part list: first, nobody is immune to cancer (whether you drink or not), second, ‘everything’ causes cancer, and third, it is hard to avoid cancer-causing risks/carcinogens. In this way Jessica constructs reducing or stopping drinking as futile given it may not make any appreciable difference to getting cancer in the future.

Other participants also described how everything causes cancer. When asked if the alcohol-cancer link was something that she discussed with her friends, Emily responded:

Just like, briefly, and we probably laugh, ‘Oh you shouldn’t drink so much you’ll get bloody cancer’, ‘Oh you get cancer from anything, you know, you get cancer from … eating enough burnt toast’. Emily, 52 years; consumes 2–3 drinks twice a week, 5+ on the weekend

Emily describes her and her friends’ laughter at the notion that alcohol might cause cancer, downplaying the moral imperative that ‘you shouldn’t drink so much’ and dismissing the ‘bloody cancer’ link by using the example of burnt toast. Toast is a mundane, everyday breakfast food, a comparison that deftly constructs everything in daily life as potentially cancer-causing. From this view, it would simply be unmanageable to reduce all cancer risks, and there is a sense of ‘risk fatigue’ such that people are tired of needing to navigate so many risks in contemporary life.

Steven expanded on the implications of ‘risk fatigue’:

Cancer is a tricky one because it so touches on so much, on sort of everyone’s life. I think, sooner or later, you feel like, I’m on a long enough timeline we’re all going to get cancer, if something else doesn’t kill us first so. It’s something I’m aware of, but I think if you if you try to go through life thinking about things that may be carcinogenic or may be increasing your risk and avoiding them at all costs, I don’t know, it could get quite debilitating. Steven, 45 years; drinks 1–2 drinks, once a week

Steven considers the temporalities of cancer risk by suggesting that if we live long enough, cancer may affect us all. He positions alcohol as just one of many carcinogens in life, and that thinking about—and trying to avoid—all these risks would make life ‘debilitating’. He positions the alcohol-cancer association as just one factor in a broad array of factors that can increase risk, enabling his conclusion—that trying to avoid all risk factors would be extremely difficult—to appear sensible and rational. Other participants also described how there are too many risk factors to avoid and for some led to them ‘blocking’ out the information about alcohol and cancer, as Maureen describes:

I think we just block it out because we don’t want to know about it because everything’s killing everyone. Maureen, 50 years; consumes 8 drinks, 2–3 times/week

Maureen’s use of ‘we’ here speaks to the normative approach of ignoring risks. Her sweeping statement that ‘everything’s killing everyone’ reinforces a sense of risk fatigue and minimises the importance of the alcohol-cancer link. If everything is a risk then knowing more simply increases worry, making it pointless to change drinking practices.

### Risk begets risk: accepting the alcohol-cancer evidence creates new risks

#### Risks to wellbeing

In undermining the link between alcohol and increased cancer risk, participants were able to construct accepting the evidence of a link as itself risky. Giving up alcohol posed immediate risks to the pleasure alcohol affords them in their daily lives and to their mental health. Some participants explained how not having the enjoyment and health-enhancing benefits of alcohol was a risk to their wellbeing. Drinking was constructed as pleasurable, fun, lovely, good, habitual and social, and therefore it was worth taking the risk of getting cancer, as Sandra explains:

I mean, I think about it, but I also don’t want to change my lovely habit of having a glass of wine, I try not to feel guilty about it. And I also know that there are people that have never touched any alcohol and also get cancer, so I sort of feel like well, it’s worth taking the risk. For the enjoyment of a glass of wine. Sandra, 52 years; consumes 1–2 drinks, 3–4 times/week

Sandra delegitimises the alcohol-cancer link by commenting on people who get cancer who do not drink, which functions to rationalise her view that drinking is worth it. She describes her glass of wine as a ‘lovely habit’ that she enjoys, but notes that knowing about the alcohol-cancer link means she experiences unpleasant feelings of guilt. However, her immediate pleasure in the glass of wine outweighs any future risk.

Deborah was able to give a real life example of this hypothetical ‘weighing up’. She discusses a friend with breast cancer, and narrates a conversation with this friend about drinking alcohol:

I’ve got a friend who’s actually got breast cancer at the moment, she is not very well. And we were kind of talking about drinking and we kind of talked and so she said, you know I wouldn’t give up all the good times, where I’ve had champagne and fun times, almost for this, like it’s like the fun times kind of almost outweigh them [pause] that makes sense to not outweigh it, but it was almost worth it having some really good fun times that you’ve associated with alcohol. Deborah, 53 years; consumes 3–4 drinks, 2–4 times/week

Here Deborah narrates how her friend seems to accept that alcohol may have played a role in her getting cancer. Nevertheless, for her friend, alcohol is aligned with ‘all’ of the ‘really good’ and ‘fun’ times, making drinking ‘almost worth it’.

Most participants positioned themselves as responsible, self-regulating citizens who used alcohol to manage busy lives and negative emotions. Within this context, knowledge about alcohol and cancer risk was constructed itself as risky, because it simply added to their guilt, worry and anxiety. Jade and Maureen describe this as follows:

It’s sort of been more recently that I’ve been aware that any degree of alcohol over a long period of time is damaging. That’s probably what added to my guilt and worry, I suppose, about having a couple of glasses of wine a few nights a week, if that could be as damaging to me as [name], that scares me. Jade, 51 years; consumes 3–4 drinks, 2–3 times/week

So, even though I know alcohol does that and is terrible for that I kind of block it out, because otherwise I’ll get anxiety. Maureen, 50 years; consumes about 8 drinks, 2–3 times/week

Jade describes multiple negative emotions (guilt, worry, fear) that she experiences around her awareness that ‘any degree of alcohol’ is damaging, which is intensified due to her personal experience of a cousin who died of cancer who was a heavy drinker. Maureen describes that knowledge about the alcohol-cancer association causes her anxiety so she blocks it out.

#### Moral risks

Many participants also constructed potential moral risks of accepting the alcohol-cancer association, both for themselves and for others. Here participants drew on the healthism discourse, but rather than positioning people who engage in risky behaviours as morally dubious (because they are not taking care of their health), they turn this around to show how it can lead to blaming individuals for their ill-health. In the case of alcohol and cancer, they firmly construct this as disrespectful and morally problematic, as Jodie’s talk demonstrates:

I think taboo is not the right word, but if somebody has got cancer and are drinking it would be, what’s the word, it would be, it would be a difficult conversation to go—do you think it has to do with your drinking? Because everybody cares about ‘I’ve got breast cancer or lung cancer’ I’ve known people that have had lung cancer that didn’t smoke. Yeah so it’s that thing and because lung cancer is associated with smoking, but what cancer is associated with drinking? It’s not it’s not a direct link, so it’s the unspoken thing you would never talk about it because it’s really disrespectful. Jodie, 52 years; consumes 2–3 drinks, 4+ times/week

Jodie’s talk highlights that linking someone’s cancer with their drinking would position both the person with cancer, and also the person suggesting it, as morally questionable. It would be ‘disrespectful’, and therefore ethically problematic, especially given the doubt participants cast on the evidence for a causal relationship between drinking and cancer. Jodie includes lung cancer in her description to emphasise her point that getting cancer, even lung cancer, is not always due to a particular ‘bad’ behaviour.

Jason and Amber also identified the risks of applying the alcohol-cancer association to a person:

I don’t know if we want to sit in judgment on people. Like I can think of friends, my mother’s [friends] who have had breast cancer, who have been heavy drinkers uh, but I would be very reluctant. I’m just trying to sort of think consciously about my thoughts here right, like I think I’d be very reluctant to think about that person in relationship to kind of cause and effect because it feels a bit rough right? Jason, 41 years; consumes 5–6 drinks, 2–4 times/week

I’m not trying to go into judgment but I’m thinking, this is, I think this is your coping mechanism for life, you know. Amber, 51 years; drinks a bottle of wine, 1–2 times a month

For Jason, applying the alcohol-cancer link to people who have had cancer is constructed as sitting in judgment. He emphasises how difficult this is to explain (‘think consciously about my thoughts here’) and how reluctant he is to do this, concluding that ultimately for him it feels ‘a bit rough’. Amber also discusses avoiding judgment when talking about her friend with bowel cancer, using a disclaimer (‘I’m not trying to go into judgment’) which shows that judgment could potentially be appropriate here. She then describes her understanding of her friend’s drinking by labelling it a ‘coping mechanism’ for everyday life.

## DISCUSSION

This study demonstrated how adults in a high-income Western society discussed alcohol and their drinking, and how they understood and considered the evidence that alcohol consumption is associated with increased cancer risk. Participants talked about knowing about this evidence, and constructed their own drinking as responsible and moderate, consistent with previous research (Muhlack *et al*. 2018, Gough *et al*. 2020, Kersey *et al*. 2022). Yet they also used a range of discursive strategies to undermine the evidence and demonstrate that accepting the evidence would give rise to additional risks. These included risks to wellbeing because drinking alcohol provided pleasure, enjoyment and relaxation, and because knowledge about cancer risks caused anxiety, worry and guilt. Further, participants constructed a moral risk of accepting that drinking alcohol increased one’s risk of cancer because it implied that cancer could be attributed to a person’s behaviour. They did not want to blame/judge a person for a cancer diagnosis, constructing such a position as morally indefensible because ‘everything causes cancer’ and there are many exceptional cases of people with cancer not having consumed alcohol.

People are bombarded with messages about ‘risky’ behaviours that should be avoided to maintain personal health (May *et al*. 2022, Lupton 2023). Public health discourses about risk tend to assume people are rational individuals who take up evidence and information and apply it in a logical manner. However, the current findings demonstrate that midlife adults take up and make sense of evidence and risk information within the context of their everyday lives and behaviours, and within competing discourses and understandings. Participants drew on their own ‘lay epidemiologies’ of risk around drinking, as others have shown (e.g. Larsen *et al*. 2023). It was not straightforward for them to apply population-level risk information to their own lives, rather this involved a range of considerations which contributed to them dismissing, undermining, ignoring and rejecting it.

### Dismissing, ignoring and undermining the evidence

The cancer risks of alcohol consumption were viewed by these midlife adults as not relevant to their own controlled, responsible and moderate patterns of drinking. They drew on risk, healthism and moderation discourses to position their drinking as undertaken with awareness of cancer risks and even good for their health and wellbeing (Crawford 2006), and constructed alcohol consumption as risky for cancer only when it went beyond specific (unnamed) levels of drinking. Drinking in moderate ways aligns with dominant framings used by alcohol companies that position themselves as facilitators of ‘responsible’ and ‘moderate’ consumption by deserving individuals (Room 2011, Goodwin and Griffin 2017, Mugavin *et al*. 2023). Use of this framing is not surprising in a country where alcohol is normalized, pervasive, and seen as a core, positive part of adulthood and social life (Espiner 2023).

Many participants also positioned cancer risks as not salient because they were sceptical about the evidence. They drew on discursive strategies to undermine the evidence, including contrasting the strong evidence for tobacco as a risk for cancer with weaker evidence for alcohol. They employed personal accounts of exceptional cases where people who had never drunk alcohol had been diagnosed with cancer, reinforcing the tenuousness of the link. Many participants also situated this as just one of many carcinogens they have to navigate in everyday life. These constructions worked to undermine the credibility of the association and justified their ongoing (moderate) drinking. Similar strategies were used by Australian adults when discussing placing labels warning of cancer on alcohol products (May *et al*. 2022). They challenged the legitimacy of alcohol-related cancer messages through discursive strategies such as undermining the scientific validity of the evidence, drawing on anecdotal evidence, providing excuses and shifting blame to others.

Some participants constructed the evidence for alcohol-related cancer risk as something they could choose to ignore or block out. This was a rational way for them to avoid feeling guilty, worried and anxious about their drinking. Foley and colleagues (Foley *et al*. 2024a) have discussed how ignorance is a ‘regular and expected feature of daily life’ (p.238), partly because there is always the potential that what we accept as current knowledge may change in the future. As health risks are in the future, and risks may not be real given personal accounts that provide contrary evidence, ignoring risk is constructed as commonsense. Previous research has found that despite knowing about the alcohol-cancer link, women at midlife may not perceive themselves as candidates for cancer; personal belief systems and experiential knowledge shaped their notions of risk (Lunnay *et al.* 2021b, Batchelor *et al.* 2023, Foley *et al.* 2024a, 2024b, 2024c).

### Worth taking the risk: factors that override cancer risks

When participants accepted that there may be some increased future risks of cancer associated with alcohol consumption, many contextualised them as being outweighed by other considerations, including the immediate benefits and pleasures of drinking. Some participants constructed drinking alcohol as enhancing their health and wellbeing in a way that outweighs long-term health risks, similar to previous research (e.g. Lyons *et al*. 2014, Larsen *et al*. 2023, Foley *et al*. 2024c, Kersey and Lyons 2025). Research shows that adults at midlife construct drinking alcohol as a wellbeing-enhancing activity that is pleasurable, fun, sociable and relaxing (Emslie *et al.* 2012, 2013, 2015, Lunnay *et al.* 2021a, 2021b, 2023, Keane 2023, Kersey and Lyons 2025).

The current findings also demonstrated how participants constructed new risks that they had to consider if they accepted the evidence on the alcohol-cancer risk, namely risks to their wellbeing and moral risks. As scholars have argued (Green 2009, Lupton 2023), risk is a pervasive concept, central to our lives and subjectivities, something that is seen as being able to be managed through individual behaviour and intervention, and is associated with ‘notions of choice, responsibility and blame’ (Lupton 2023, p.33). Participants were very reluctant to blame people with cancer for their illness, which could potentially occur if they accepted that alcohol use was associated with cancer risk. A healthism discourse situates avoiding health risks as a moral issue, but participants rejected this because it could lead to judging friends and family which breaches conventions of friendship and support. This may be a form of ‘relational healthism’ in which practices related to healthism, such as individual control and discipline, are at odds with relationship norms such as support and acceptance (Robson *et al*. 2023).

### Implications for public health and government messaging

Public health messaging about the health risks of alcohol prioritises long term health over shorter term pleasures and other values (Nicholls and Hunt 2025). Much public health alcohol research has focused on harms, while public health discourse has downplayed pleasure ‘as either marginally significant or as a kind of misperception driven by external forces including marketing, custom, social norms and peer pressure’ (Nicholls and Hunt 2025, p.1075). Drawing on a tranche of sociological research, Pennay and Livingston (2025) argue that it is not justifiable to treat long-term health as more important than short-term pleasures. The current findings that other factors override cancer risk considerations for many midlife adults emphasize the importance of taking people’s own meanings and sense-making into account. If public health messaging fails to consider how people understand their drinking, and the additional risks that are created by public health advice, then messages are unlikely to resonate with populations and may have unintended negative consequences (e.g. blaming people for their illnesses).

Sophisticated discussions around risk, pleasure, and alcohol are also needed at the policy level (Nicholls and Hunt 2025, Pennay and Livingston 2025). Government low-risk drinking guidelines recommend a maximum number of drinks per occasion or per week. However, government campaigns often promote industry-favoured ‘responsible drinking’ messages, which focus on short-term harms such as dependence, anti-social behaviour, and drink-driving, and frame a small number of problem drinkers as causing most of the harms. These pay little attention to associations between relatively low levels of alcohol consumption, cancer and heart health (Petticrew *et al.* 2018). Together, concepts of ‘responsible drinking’ and maximum recommended levels convey the notion that some levels of alcohol consumption are without risk. Confounding this further, public health understandings and approaches do not factor in pleasure or fun, which leaves pleasure to the alcohol industry to co-opt for its commercial gain (Nicholls and Hunt 2025, Pennay and Livingston 2025).

Subjective concepts of moderate and responsible drinking cover a range of drinking amounts and often fit within socially acceptable and economically valorized levels of consumption (FARE 2020, Kersey *et al*. 2022, Mugavin *et al*. 2023, Davies *et al*. 2024). For many midlife adults, drinking is a source of pleasure and fun, provided the harms are not immediate or obvious. As Nicholls and Hunt point out, public health operates ‘in a culture that both encourages and condemns the pursuit of hedonic pleasures’ (p.1077). How can public health researchers and policy-makers incorporate such complexities into their work? Research that examines people’s uptake, resistance, and negotiation of health promotion messages is crucial. We need greater nuance and sophistication around communicating the health risks of alcohol. Acknowledging that people weigh up multiple risks alongside the perceived benefits will enable public health messages to be more relatable and accessible. Importantly, such messages may fall differently across different sectors of populations. The immediate pleasures afforded by drinking alcohol may be rational if futures are uncertain due to poverty, discrimination, and other disadvantages (Nicholls and Hunt 2025).

Public health messaging has become more challenging since the Covid-19 pandemic. Many people increasingly challenge expert knowledge and seek advice from alternative sources, which means that high trust is required in both experts and the health information they provide (Foley *et al*. 2024b). Changing government advice around alcohol has already led to scepticism about government messaging, particularly where it does not accord with ‘commonsense’, so it is important to consider how expert health information can retain its legitimacy with target audiences (Meyer *et al*. 2019, Davies *et al*. 2022). Educational campaigns may be useful if they are viewed as relevant and trustworthy in the lives of different cohorts (Bujalski 2024). The current sample may feel they know more than experts or may be more sceptical of the evidence than others due to their education and relatively privileged positions.

### Limitations

Study participants were relatively homogenous, with more females than males, mostly NZ Pākehā/European, heterosexual, and well-educated. They had previously participated in a survey about alcohol and health outcomes, including cancer, so had already considered this topic. The context of talking to a researcher about drinking practices and risk shaped the co-constructed data, including what participants said, the discursive strategies they used, and how they presented and positioned themselves. Nevertheless, findings and theoretical insights may help with developing future research on how scientific consensus on alcohol-related cancer risks comes with other risks, and how to develop messaging that resonates with adults at midlife.

## CONCLUSIONS

This study contributes insights into why people do not change their drinking behaviours even when they are aware of evidence that alcohol is a carcinogen. Adults at midlife draw on a number of strategies to minimize, dismiss, or ignore this evidence, and this needs to be considered in the development of public health campaigns. Importantly, the most effective way to reduce population-level alcohol consumption is by changing the alcohol environment through policies that address the availability, accessibility, and acceptability (marketing and promotion) of alcohol (Babor *et al*. 2022) in culturally and gender-responsive ways (WHO 2024). Currently, industry advertising and corporate social responsibility messages are pervasive and function to embed alcohol as a desirable, pleasurable, and normalized product in society in ways that contribute to alcogenic environments and legitimize ‘responsible drinking’ (Davies *et al*. 2023, Szmigin 2024). Public health messaging around the long-term health risks of alcohol consumption competes in this space and is often resisted or ignored by target populations because it creates additional risks and fails to take pleasure into account.

## Data Availability

The data used in this article cannot be shared publicly because participants consented to only the research team having access to their interview transcripts.
